# *Ascophyllum nodosum* Based Extracts Counteract Salinity Stress in Tomato by Remodeling Leaf Nitrogen Metabolism

**DOI:** 10.3390/plants10061044

**Published:** 2021-05-21

**Authors:** Emilia Dell’Aversana, Valerio Cirillo, Michael James Van Oosten, Emilio Di Stasio, Katya Saiano, Pasqualina Woodrow, Loredana Filomena Ciarmiello, Albino Maggio, Petronia Carillo

**Affiliations:** 1 Department of Environmental, Biological and Pharmaceutical Sciences and Technologies, University of Campania “Luigi Vanvitelli”, Via Vivaldi 43, 81100 Caserta, Italy; emiliadellaversana@gmail.com (E.D.); katyasaiano@libero.it (K.S.); pasqualina.woodrow@unicampania.it (P.W.); loredanafilomena.ciarmiello@unicampania.it (L.F.C.); 2 Department of Agricultural Sciences, University of Naples “Federico II”, 80055 Portici, Italy; valerio.cirillo@unina.it (V.C.); dr.m.vanoosten@gmail.com (M.J.V.O.); emiliodistasio@gmail.com (E.D.S.); almaggio@unina.it (A.M.)

**Keywords:** seaweed extract, biostimulants, abiotic stress, salinity, osmolytes, minor amino acids, osmolality, salt tolerance

## Abstract

Biostimulants have rapidly and widely been adopted as growth enhancers and stress protectants in agriculture, however, due to the complex nature of these products, their mechanism of action is not clearly understood. By using two algal based commercial biostimulants in combination with the *Solanum lycopersicum* cv. MicroTom model system, we assessed how the modulation of nitrogen metabolites and potassium levels could contribute to mediate physiological mechanisms that are known to occur in response to salt/and or osmotic stress. Here we provide evidence that the reshaping of amino acid metabolism can work as a functional effector, coordinating ion homeostasis, osmotic adjustment and scavenging of reactive oxygen species under increased osmotic stress in MicroTom plant cells. The Superfifty biostimulant is responsible for a minor amino acid rich-phenotype and could represent an interesting instrument to untangle nitrogen metabolism dynamics in response to salinity and/or osmotic stress.

## 1. Introduction

The exponential growth in the world’s population and the improved quality of life is expected to double food demand by 2050 [1]. Agricultural intensification will be a key factor for expanding food supply in the next years. However, the overuse of fertilizers, water and pesticides that started with the Green Revolution can no longer be pursued due to the negative impacts on the environment [2]. In the last decades it has become clear that increasing the use of chemicals in agriculture does not always correspond to an increase in productivity [3,4]. The shallow root systems of many crop plants have a limited mineral uptake capacity. Therefore, most of the applied mineral fertilizers are lost in the soil, causing pollution, salinization and soil degradation [5]. This is particularly critical for the greenhouse industry, which covers an area larger than 700,000 ha in the world [6], and whose fertilizer and water inputs per unit area are much higher than any other agricultural system [7]. In particular, nitrogen (N) inputs represent not only the highest cost for farmers [8], but also a major cause of environmental pollution and health risks [9,10,11,12]. More than 50% of N fertilizers applied is lost in the soil–plant system, causing the contamination of surface and groundwater as well as release of air pollutants and greenhouse gases [8]. Therefore, sustainable agricultural intensification can be achieved only by improving resources use efficiency (RUE) while reducing the use of chemical fertilizer and, in particular, nitrate [13]. It will also require that the use of saline and recycled water for irrigation [1,14,15,16]. It is important to verify if the reduction in the use of N-fertilizers can affect the pool of nitrogen-containing compatible compounds. These latter are synthetized in plants, also in response to abiotic stresses (e.g., salinity, drought, high light, etc.), and interact with molecules and structures. This acts to preserve the activity of macromolecules and maintain the integrity of membranes against stresses and scavenging ROS [17,18]. The need to improve RUE and reduce environmental pollution while facilitating the uptake and assimilation of nutrients has led to the adoption of biostimulants as an organic alternative in agriculture [19,20,21,22]. Biostimulants have been defined by du Jardin [23] as natural substances or microorganisms that, when present in the plant environment, can improve plant growth even under abiotic stress conditions such as salinity or nutrient deficiency, enhancing nutrient availability, uptake and assimilation. Natural biostimulants are classified neither as pesticides nor fertilizers because they do not have direct effects on pests and pathogens or supply large amounts of nutrients. They represent a promising strategy to guarantee food safety without adversely affecting the environment [24]. In particular, algae and their extracts, which have been used in agriculture for centuries to improve plant growth and resistance to biotic and abiotic stresses, represent an interesting category of biostimulants for agriculture. Brown algae, which includes about 2000 species, and in particular *Ascophyllum nodosum*, *Fucus* spp., *Laminaria* spp., *Sargassum* spp. and *Turbinaria* spp. [25] are mainly used in agriculture as biofertilizers [26]. Several studies have demonstrated the innumerable benefits conferred by their application, such as enhancement of seed germination, improvement of crop yield, induced tolerance to biotic and abiotic stresses, and the extension of post-harvest [27]. In addition to macro and microelements, these extracts also contain amino acids, vitamins, hormones, alginates and various polysaccharides that have effects on metabolism of treated plants. These act as promoters of growth and crop yield [28,29,30,31,32,33,34]. Among the various actions of algal extracts, it has been found that they can improve the plant tolerance to salinity [19,35,36]. Salinity is able to exert adverse effects on plants by decreasing plant’s ability to extract water from the soil and the accumulation of ions like Na^+^ and Cl^−^ at toxic concentrations in cell tissues. This dual osmotic and ionic stress can reduce cell expansion, inhibit tissue growth, and cause nutritional imbalances and oxidative stress. All of these factors can strongly decrease plant growth, development and survival [37,38]. Plants try to cope with salt stress by triggering specific strategies to increase cellular osmotic control, restoring cellular and water homeostasis in order to minimize stress damage and reactivate growth [39,40]. Algal extracts can promote plant defense responses by eliciting the synthesis of protective compounds that may act as compatible osmolytes and antioxidants. These compounds are also able to stabilize membranes and proteins, scavenge ROS, buffer cellular redox potential [27,36,41]. *Ascophyllum nodosum* extracts are widely used in commercial formulations. They have been shown to decrease Na^+^ accumulation in leaves and the net uptake of Na^+^ in roots [36,42]. They are rich in betaines which act as compatible osmolytes [31], helping the plant to overcome osmotic stress. Due to the extremely complex nature of the algal extracts, many studies are currently focused on understanding which components of these complex formulations have a direct effect plant metabolism [19,27,35]. Di Stasio and coworkers [27] analyzed the composition and effects of two commercial formulations of *Ascophyllum nodosum* based extracts, Rygex and Superfifty on fruits of MicroTom tomato plants grown under nutrient deficiency and salt stress. The study found that Superfifty contained very high levels of mannitol and sorbitol, which could have been able to exacerbate the symptoms of osmotic stress instead of being beneficial under salinity [43]. The Superfifty biostimulant was able to stimulate plant growth and increased the nutritional value of tomato fruits in non-stress conditions. In fact, while endogenous mannitol is a compatible solute for plants, if supplied exogenously it can reduce the water potential in the soil medium rendering it difficult for water and nutrients uptake by the plant [44].

Several studies have been performed to assess the effects of seaweed based extracts on fruit metabolism [27,35,45,46,47,48,49], none of these have focused on changes in amino acid and carbon metabolism in leaves as a key mechanism affected by these biostimulants. Therefore, the aim of the present work was to assess the putative effects of *Ascophyllum nodosum* based extracts on nitrogen and carbon metabolism of MicroTom plants under salinity. Our results may be useful to provide an understanding of the regulatory mechanisms elicited by these plant biostimulants in the control of plant metabolism, which constitutes a prerequisite for the standardization of use of these products. For our experiments we used leaves of plants submitted to the same experimental design of Di Stasio and coworkers. We analyzed the growth parameters in relation to the leaf ion content, carbohydrates, proteins, amino acids, and antioxidant metabolites. In particular, the remodeling of leaf amino acid profile was investigated focusing on its possible role in plant tolerance.

## 2. Results

### 2.1. Biometric Traits

**Figure 1:**
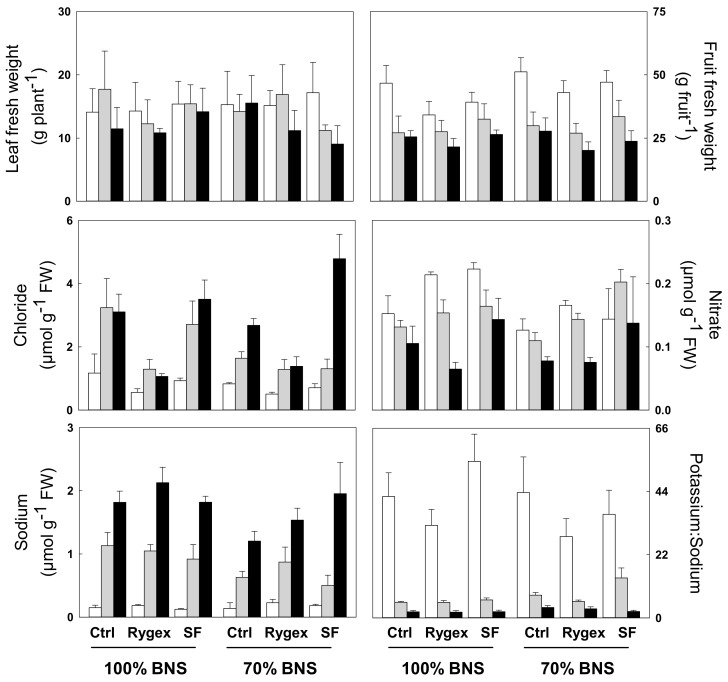
Leaf fresh weight, fruit fresh weight, leaf content of chloride, nitrate, sodium ), and the potassium to sodium ratio. MicroTom plants were harvested at 90 days after sowing (DAS) after being fertigated since 35 DAS with a 100% or 70% basic nutrient solution (BNS), in combination with three salinity levels of 0 (white bars), 42.5 (grey bars), and 85 (black bars) mM NaCl and two different commercial two commercial *Ascophyllum nodosum* extracts, Rygex at 2.5 mL L^−1^ and Superfifty (SF) at 2.0 mL L^−1^. Control (Ctrl) was without biostimulant. The values are mean ± S.D. (*n* = 5 for morphological traits and *n*=3 for the ion measurements). Significance of the main factors (nutrient solution, biostimulant and salinity) and their interactions is shown in Table 1 and Table 2.

The biometric traits of each nutrient, salt stress, and biostimulant were collected with the aim to evaluate basic growth and fruit production with respect to each treatment. Significant differences were observed for leaf fresh weight (FW) only under 85 mM NaCl (S2), that is a decrease of 21% with respect to Control (Table 1, Figure 1). Salinity significantly decreased fruit FW (as previously reported in Di Stasio and coworkers [27]), and dry weight (DW) both at 42.5 mM NaCl (S1) and S2 but did not affect the number of fruits. Rygex biostimulant (B) significantly (*p* < 0.01) reduced the fruit FW (−17% compared to Control) [27].

### 2.2. Leaf Mineral Profile

In order to understand the effects of nutrient solution and biostimulant treatment with regard to salt tolerance, the leaf mineral profile was analyzed in order to understand how each treatment affected ion uptake and accumulation. The salinity treatments had the most pronounced effect on the leaf content for all minerals analyzed followed by the two other tested factors: biostimulant (B) and nutrient solution (BNS) (Table 2, Figure 1). The nutrient solution at 70% (70% BNS) significantly increased magnesium (+21%), while reduced nitrate (−13%) and sodium (−22%) compared to respective values at 100% BNS (Table 2, Figure 1). The treatment with Rygex was the only factor with a significant effect on leaf Cl^−^ content, which was found 52% and 56% lower than that of control and Superfifty treatments, respectively. However, Rygex was also able to significantly increase Na^+^ concentration (+18%) compared to the Control, negatively affecting the potassium to sodium ratio, which decreased of 27% compared to Control (Table 2, Figure 1). Nitrate content was significantly (*p* < 0.05) higher in Superfifty than in the other two treatments (0.17 versus 0.12 and 0.14 mg g^−1^ FW). Magnesium was significantly enhanced by Superfifty (+21% compared to Control), too (Table 2). The salinity factor had a significant effect on the ions content, determining the accumulation of both toxic elements Cl^−^ (+2.4- and 3.5-fold higher than Control in S1 and S2, respectively) and Na^+^ (+5- and 10-fold higher than Control in S1 and S2, respectively). Potassium significantly decreased only under S2 (−32% compared to Control); while the potassium to sodium ratio strongly decreased both under S1 and S2 compared to Control (7.41 and 2.54 versus 39.47). Nitrate was highly affected by salinity, too. It slightly but significantly decreased (−12% compared to Control) under S1, while the decrease under S2 was much stronger (−42% compared to Control) (Table 2, Figure 1).

### 2.3. Antioxidant Metabolites

As antioxidant metabolites play a major role in salt tolerance, the activity of six antioxidants were measured to better understand the role of each treatment under salinity stress. ANOVA highlighted that the N × B interaction was highly significant (*p* < 0.001) for dehydroascorbate (DHA) and DHA to ascorbate (Asc) ratio, with the highest values recorded for Superfifty (Table 3, Supplemental Figure S1). DHA did not significantly change in control and Rygex treatments but more than doubled under Superfifty treatment. However, salinity strongly decreased both DHA and DHA to Asc ratio to values 30% lower than Control, while Asc remained unchanged. The treatment 70% BNS significantly decreased glutathione (GSH) concentration, while Superfifty strongly increased it (+60% compared to Control) while decreasing reduced glutathione (GSSG) to GSH ratio (−41% compared to Control) (Table 3, Supplemental Figure S1).

### 2.4. Carbohydrates and Soluble Proteins

Total carbohydrates were analyzed in order to understand the role of each treatment in regard to carbon assimilation. Similarly, total soluble proteins were evaluated to understand the effects of each treatment on nitrate assimilation. ANOVA highlighted that the N × B × S interaction was highly significant (*p* < 0.001) for all analyzed carbohydrates except fructose, with the lowest values recorded for Rygex and Superfifty under S0 and S1 × 100% BNS compared to Control (Table 4, Supplemental Figure S1). Fructose was the only sugar which slightly but significantly decreased under 70% BNS (−12%). The treatment with both biostimulants significantly decreased glucose, hexoses, sucrose, starch and soluble proteins (Table 4). Whereas, salinity significantly increased fructose (+28% on average under S1 and S2), sucrose (+21% under S2), starch (+23% under S1) while decreased glucose (−15%) compared to respective controls (Table 4, Supplemental Figure S1).

### 2.5. Total Free Amino Acids Content

**Figure 2:**
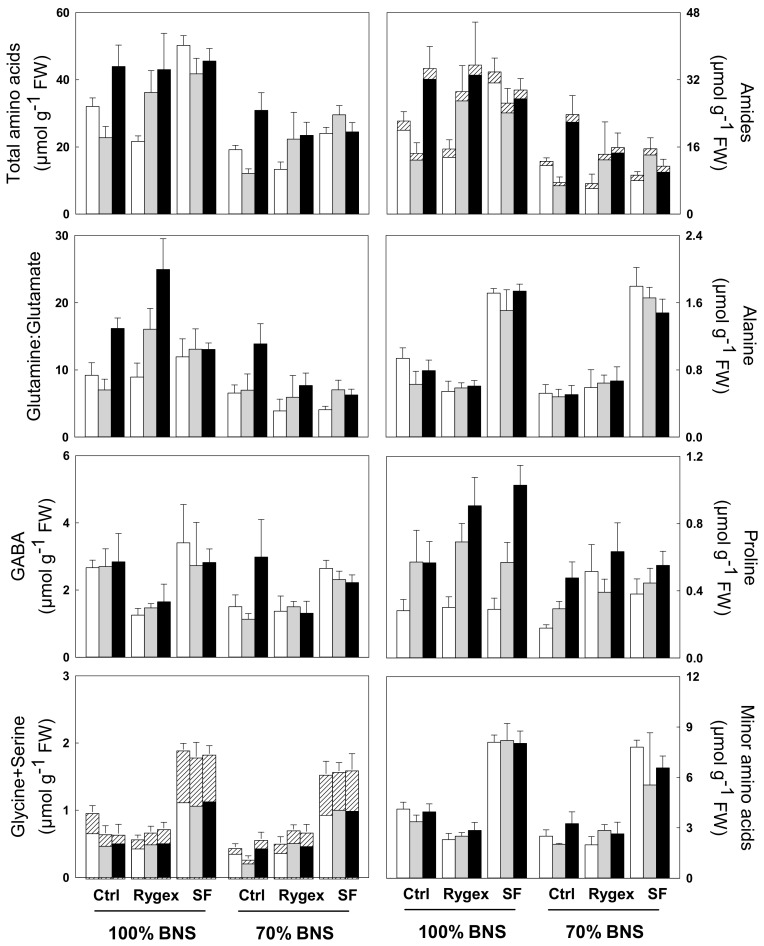
Leaf content of total amino acids, amides (the black and white fine pattern in the bar represents only asparagine), glutamine:glutamate, alanine, GABA, proline, glycine + serine (the black and white fine pattern in the bar represents only glycine), minor amino acids. Bar colors and experimental design are as in Figure 1. The values are mean ± S.D. (*n* = 5)). Significance of the main factors (nutrient solution, biostimulant and salinity) and their interactions are shown in Table 4 and Table 5.

The nutrient solution (BNS) had the most pronounced effect on the free amino acids content followed by B treatment and salinity (Table 5, Figure 2). At 100% BNS glutamine, GABA, glutamate alanine and asparagine were quantitatively the most abundant amino acids in MicroTom leaves accounting for 65.8%, 6.4%, 5.2%, 2.7% and 2.3% of total free amino acids. The 70% BNS condition significantly decreased total amino acids (37.47 versus 21.99 µmol g^−1^ FW) and all the free amino acids, in particular glutamine (24.64 versus 11.74 µmol g^−1^ FW), compared to the respective values at 100% BNS. However, under 70% BNS the relative percentage of free amino acids in relation to total amino acids changed, in fact GABA, glutamate alanine and asparagine accounted for 53.4%, 8.5%, 8.0%, 4.1% and 3.5%. Moreover, also the relative percentage of minor amino acids compared to total amino acids increased at 70% BNS compared to 100% BNS (17.7% versus 12.9%) (Table 5, Figure 2).

The treatment with Rygex significantly decreased GABA (−38%) and minor amino acids (−21%), while increased that of proline (+46%) compared to respective controls. The treatment with Superfifty was able to induce a significant increase of all amino acids except for glutamine, and the most consistent increases with respect the controls were those of alanine (+2.57 fold), glycine (+4-fold), serine (+2.41 fold), threonine (+2.84-fold) and minor amino acids (+2.3-fold) (Table 5). The total free amino acids content, glutamine and proline significantly increased under S2 salinity (+32%, +54% and +115%, respectively) compared to respective controls. Proline content increased 56% also under S1 compared to Control (Table 5, Figure 2). ANOVA highlighted that the N × B × S interaction was significant for alanine and minor amino acids, whose content strongly increased under Superfifty treatment independently of salinity and BNS compared to controls; glutamine that increased under Superfifty × S0/S1 × 100% BNS; proline, whose content increased under B treatments × S2 × 100% BNS compared the respective controls; total amino acids that increased compared to control treatment under Rygex × S1 × 100% BNS and Superfifty × S0/S1 × 100% BNS, except fructose, with the lowest values recorded for Rygex and Superfifty under S0 and S1 × 100% BNS compared to Control (Table 5, Figure 2).

### 2.6. Leaf Osmolality

Leaf osmolality is considered a reliable means of measuring the concentrations of osmotic substances in plant tissue. Osmolality typically increases in response to salt stress, with more tolerant plants accumulating larger amounts of compatible solutes. Osmolality of leaves was measured for each treatment. MicroTom sap osmolality was, on average, 324 and 356 mOsmol kg^−1^ in leaves of S0 plants under 100% and 70% BNS, respectively, independently of SWEs. The sap osmolality significantly increased in all S2 plants compared to S0 treatments (Table 6). Potassium was the main ion contributing to osmolality independent of salinity, with the exception of Superfifty × S2 treatments in which all ions equally contributed. The relative contribution of the main inorganic ions to osmolality was, on average, 56% and 50% in 100% BNS and 70% BNS, respectively. In particular, under 100% BNS it was significantly lower than average only in SWEs × S2 treatments, while under 70% BNS it was significantly higher than average in Rygex × S0 treatment. In 70% BNS, it was also evident that the relative contribution of the main inorganic ions was significantly higher in SWEs treatments than in Control ones. Under 100% BNS, the measured organic osmolytes (soluble sugars and free amino acids) contributed to osmolality mainly in Control and Superfifty × S0 (30.2% on average), while under 70% BNS, Rygex showed a contribution similar or also higher than other two conditions. The contribution of soluble sugars to osmolality was higher in Control × S0 and S1 under 100% BNS and Control × S1 and S2 and Rygex × S0 and S1 under 70% BNS than in all other treatments. The sugar content remained almost unchanged in all other treatments, being on average 12.5% independently of the two fertilization treatments. The amino acid contribution to osmolality that was only observed in Superfifty × S0 under 100% BNS, was mainly due to glutamine content. However, also other amino acids like alanine, minor amino acids and GABA contributed in particular in Superfifty treated samples. According to Puniran-Hartley et al. [50], other ions and metabolites present in the cell could contribute to osmolality, and their relative contribution can be calculated as difference between the total sap osmolality and the contribution of the measured major inorganic ions and osmolytes as shown in Table 6. The other osmolytes calculated contribution was on average 20% and 30% in 100% BNS and 70% BNS, respectively.

### 2.7. Principal Component Analysis

**Figure 3:**
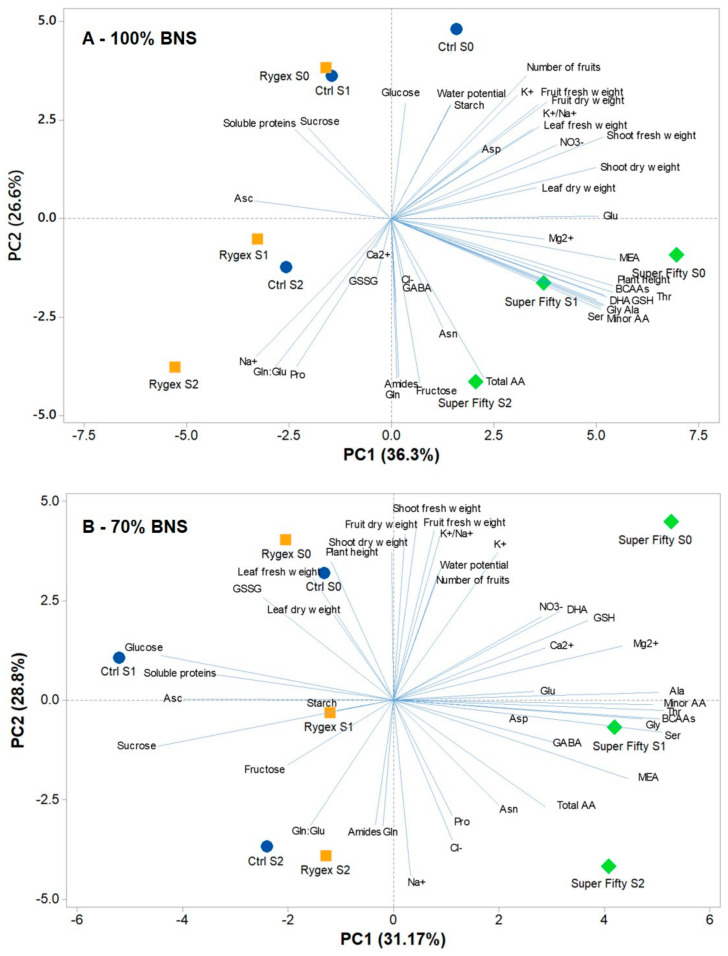
Principal component analysis (PCA) of morphological, physiological and biochemical parameters of MicroTom tomato plants harvested at 90 DAS after being fertigated since 35 DAS with a 100% (**A**) or 70% (**B**) basic nutrient solution (BNS), in combination with three salinity levels of 0, 42.5, and 85 mM NaCl (S0, S1, and S2, respectively) and two different commercial *Ascophyllum nodosum* extracts (Rygex at 2.5 mL L^−1^ and Superfifty at 2.0 mL L^−1^).

To obtain an in-depth overview of all the analyzed parameters in MicroTom tomatoes under the different treatments, a principal component analysis (PCA) was performed. Under the treatment 100% BNS, the first four principal components (PCs) were related with eigenvalues higher than 1 and explained 88.6% of the total variance, with PC1, PC2, PC3 and PC4 accounting for 36.3%, 26.6%, 16.1% and 9.6%, respectively; while, under the 70% treatments, the first four PCs, related with eigenvalues higher than 1, explained 83.9% of the total variance, with PC1, PC2, PC3 and PC4 accounting for 31.7%, 28.8%, 13.0% and 10.4%, respectively (data not shown). Under both nutrient strengths, the biostimulant treatments contributed to the clear separation on PC1, while the salt treatment contributed to the separation on PC2 (Figure 3). Superfifty treatments were concentrated in the positive side of PC1, while control and Rygex treatments were in the negative side of PC1 with the exception of Control S0 under 100% BNS. In particular, under 100% BNS, Control S0 was in the right upper quadrant, Rygex S0 and Control S1 were in the left upper quadrant, Rygex S1 and Rygex S2 and Control S2 were in the lower left quadrant, and all the Superfifty treatments were in the lower right quadrant (Figure 3A). Under 70% BNS, the treatments maintained the same quadrant distribution, with the exception of Superfifty S0 that was in the upper right quadrant and Control S0 that was present in the left upper quadrant (Figure 3B). Under 100% BNS, PC1 was positively correlated to serine, glycine, threonine, alanine, total amino acids, minor amino acids, DHA, GSH, BCAAs and MEA. PC1 was also negatively correlated with soluble proteins, sucrose and ascorbate. PC2 was positively correlated with Na^+^ and proline, while it was negatively correlated to K^+^, fresh weight, nitrate, starch, water content, aspartate and glucose (Figure 3A). Under 100% BNS, Superfifty S0 was correlated with the highest content of glutamate, MEA, minor amino acids and plant height. Superfifty S0 and S1 treatments were also correlated with the highest GSH and DHA, minor amino acids, in particular BCAAs, alanine, glycine, threonine and serine, while SFS2 with highest concentration of fructose and total amino acids. Control S0 was correlated with glucose, water content and starch. In the negative side of PC1, Rygex S1 and Control S2 were correlated to ascorbate, Rygex S0 and Control S1 with soluble proteins and sucrose (Figure 3A). Under 70% BNS, Superfifty S0 treatments contributed to produce leaves with the highest GSH and DHA, Ca^2+^, Mg^2^ and nitrate, while Superfifty S1 treatment was correlated to the highest amount of minor amino acids, BCAAs, alanine, glycine, threonine, serine and GABA (Figure 3B).

## 3. Discussion

In a previous paper of Di Stasio et al. [27], MicroTom plants were submitted to a factorial combination of two nutrient strengths (100% or 70% basic nutrient solution, BNS), three salinity levels of 0 (S0), 42.5 (S1), and 85 (S2) mM NaCl and two different commercial *Ascophyllum nodosum* extracts (Rygex at 2.5 mL L^−1^ and Superfifty at 2.0 mL L^−1^) in order to study the ameliorative effects of these two SWEs extracts on plant growth and yield. However, the study highlighted, by characterization of the SWEs, via gas chromatography–mass spectrometry analysis (GC-MS), that Superfifty contained high levels of mannitol and sorbitol. Exogenous application of these to the soil medium would likely contribute to osmotic stress [43]. Nevertheless, Superfifty was able to stimulate plant growth of 13% under a full-strength nutrient solution (100% BNS), independently of salinity, while it did not decrease growth under reduced nutrient strength (70% BNS). Both biostimulants were able to increase the nutritional value of tomato fruits, enhancing the content of minerals, antioxidants and essential amino acids but not sugars [51]. Though these data are in agreement with those obtained by Flores et al. [52] and Yin et al. [53], they should be considered in light of the fact that MicroTom plants have not only mutations in the *DWARF* and self-pruning genes, but also a third mutation, responsible for internode length, which may have pleiotropic effects on metabolism of the fruits [52,54]. The complexity of the metabolic landscape across tissues and growth stages has been recently addressed by Li et al. [55] in MicroTom, indicating that this model species is a useful resource to study metabolic regulatory networks in tomato.

While numerous studies have been published on the effects of these two biostimulants in terms of growth and stress tolerance, it is important to note that there is little focus in the literature on the composition and potential mechanisms by which algal extracts affect plants. Rygex is commonly used in agriculture, but to date only one study has looked at its composition, primarily focusing on mineral content and auxin-like effects [56]. No studies have been published to date examining the changes in nitrogen metabolism involving Superfifty. In order to understand how plants could cope with salinity under Superfifty and Rygex treatment under salinity, we analyzed the leaves of the plants treated as in Di Stasio et al. [27]. We examined the growth parameters in relation to the profile of minerals, carbohydrates, proteins, amino acids, including essential amino acids and antioxidant metabolites. In particular, we focused on the changes of sugars and amino acids in light of understanding if they could contribute as osmolytes to cell osmolality and/or could have a different protection role.

The different conditions to which plants were submitted did not cause significant biometric changes. However, Superfifty biostimulant improved plant antioxidant defense and nitrogen use assimilation increasing GSH, nitrate, alanine and minor amino acids contents. An increase in GSH, and a concomitant reduction of GSSG to GSH ratio, can prevent and/or repair damages caused by oxidative stress under salinity [57]. Moreover, the improved nitrate uptake capacity of plants and/or the remobilization of N reserves from roots both as nitrate and as amides and their translocation to shoot can improve plant nutritional status and yield [5]. The higher availability of nitrate can contribute to the synthesis of amino acids involved in salt tolerance [58]. The increased minor amino acids, and in particular BCAAs (not shown), can function both as compatible osmolytes and as alternative electron donor to fuel the mitochondrial electron transport chain (mETC) via ETF/ETFQO during starvation [59,60]. It has been found that BCAAs are also able to reduce oxidative stress in rats and prolong their survival with advanced liver cirrhosis by a not as yet characterized mechanism [61]. Previous studies have shown that changes in minor amino acids are coordinated with an increase of carbohydrates and/or amides [62,63]. In particular, it could be related to the glutamine to glutamate ratio by a control of the downstream carbon/nitrogen metabolism [64], to the low level of nitrate and high levels of hexoses [63] and sucrose [65], or to protein degradation [66]. Genes involved in minor amino acids synthesis are sugar-responsive [15]. However, this was not observed in our study, because hexoses and sucrose decreased and not increased in Superfifty treatments under salinity, glutamine (and therefore amides as sum of glutamine and asparagine) increased under S0 and S1 but not under S2, while glutamine to glutamate ratio did not significantly change compared to Control. Moreover, minor amino acids did not only potentially function as compatible metabolites and antioxidant in leaves [67], but, exported to fruits, contributed to increase the content of essential amino acids, nutritional value and antioxidant properties of tomato fruits [27] potentially improving shelf life [68].

As shown also by PCA, the most interesting effects were exerted by Superfifty biostimulant that was able to completely reshape the free amino acids profile of treated plants independently of salinity and BNS (Table 5, Figure 1). In particular, in all treatments, in addition to minor amino acids, also alanine, glycine and serine were highly increased compared to the other two treatments, independently of nutrient strength (Figure 3). The high contents of glycine and serine, independently of salinity treatment could be probably due to the presence in the Superfifty extract of high concentrations of sorbitol and mannitol [27], which induced osmotic stress and partial closure of stomata, triggering photorespiration [43]. Alanine could work in these conditions as an important mechanism of biochemical pH state, since its synthesis from pyruvate, deriving by the decarboxylation of malate by malic enzyme activity, is a proton-consuming reaction able to buffer cytosolic acidosis [69]. Moreover, using alanine, instead of amides as N storage, is less expensive for plants when ATP is low [70]. Finally, this amino acid can be again re-transaminated to pyruvate and converted to acetyl-Coenzyme A in the mitochondrial matrix to increase the production of ATP to sustain plant metabolism [71]. In all, Superfifty conditions, even if not always significantly at 70% BNS, GABA and proline were also accumulated probably to function as osmolytes and ROS scavengers to cope with salinity and/or water stress caused by the high sorbitol and mannitol content of the biostimulant [72]. Carillo [17] suggested that the synthesis of GABA by glutamate decarboxylase (GAD) could cause the dissipation of excess of energy and release of CO_2_, allowing a restart of the Calvin cycle and a lower pressure on photosynthetic electron chain that could decrease ROS generation and photodamage under osmotic stress. In addition, in case of relief of stress, after exerting function in cell protection, GABA and proline can be broken down to provide carbon, nitrogen and energy to repair damages induced by the stress [73,74].

As mentioned above, the treatment with Rygex caused a lower acquisition of Cl^–^ ions in leaves of MicroTom tomato plants, regardless of fertilization, but also a decrease of water potential [27] notwithstanding Na^+^ concentration strongly increased under salt stress. This was probably due to the fact that the increase of cellular Na^+^ was not able to interfere with K^+^ translocation from roots to shoots and with its transport through the plasma membrane, and therefore there was not a significant decrease of cytosolic K^+^ in all plants treated with both biostimulants as found in Bougainvillea under salinity [37]. Na^+^ concentration in plant tissues can also increase while cytosolic K^+^ concentration is maintained at a constant level or even increased by the release of vacuolar K^+^ [75]. The compartmentalizing of Na^+^ in the vacuole osmotically balanced by K^+^ in the cytosol could be another essential mechanism for MicroTom salt tolerance [76]. As also demonstrated by the high contribution of K^+^ to total osmolality (Table 2), the increase in K^+^ concentration in the cytosol could be enough to balance the mesophyll cells without needing the action of other metabolites, since the cytosol, the cytosolic compartments (including cytosol and hyaloplasmic organelles) and vacuoles occupy about 4–12%, 15–32% and 68–85%, respectively, of the cell volume according to Koffler et al. [77] and references therein.

## 4. Materials and Methods

### 4.1. Greenhouse Conditions, Plant Material and Experimental Design

For this experiment the cultivar MicroTom (*Solanum lycopersicum* L.), a dwarf phenotype with small red ripened fruits produced by Scott and Harbaugh [78] by crossing Florida Basket and Ohio 4013-3 cultivars, was chosen as model plant. The growth of MicroTom plants was carried out from May to August 2014 at the experimental station of the University of Naples Federico II, South Italy (lat. 43°31′ N, long. 14°58′ E; alt. 60 m above sea level), as reported by [27]. In particular, tomato seeds, germinated in peat, were grown until the third to fourth true leaf stage before transplanting in 10-cm plastic pots containing pure peat moss (100%) and drip fertigated from 35 days after sowing (DAS). The irrigation water had pH and electrical conductivity (EC) of 7.3 and 0.58 dS m^−1^, respectively. A basic nutrient solution (BNS) was used for all treatments: (mM) NO_3_^−^ = 1.93, P_2_O_5_ = 2.53, K_2_O = 7.64, MgO = 1.48 and (μM) B = 37, CuEDTA = 0.84, Fe DTPA = 10, Mn EDTA = 3.45, Mo = 2.08, Zn EDTA = 0.83, having an EC of 1.60 dS m^−1^. The BNS was supplied at two different concentrations (100 and 70% BNS) and in combination with three NaCl levels of 0, 42.5, and 85 mM NaCl (S0, S1, and S2, respectively). The MicroTom tomato plants were grown in pots and treated with two commercial *Ascophyllum nodosum* extracts, Rygex (Agriges S.R.L., Benevento, Italy) and Superfifty (BioAtlantis Ltd., Kerry, Ireland), applied as soil drench every 2 weeks until the end of the experimental period (90 DAS). Control plants were not treated with biostimulants (control). The commercial biostimulant solution, prepared with 2.5 mL Rygex L^−1^, contained 26.3 mg L^−1^ of free amino acids, of which 9.45 mg L^−1^ (26.4%) of minor amino acids, 0.1 mg L^−1^ of carbohydrates, 0.36 mg L^−1^ of organic acids, 0.42 mg L^−1^ of fatty acids and 425 mg L^−1^ of mineral nutrients. The commercial biostimulant solution, prepared with 2.0 mL Superfifty L^−1^, contained 6.28 mg L^−1^ of free amino acids, of which 0.23 mg L^−1^ (3.6%) of minor amino acids, 3.53 mg L^−1^ of carbohydrates, 2.67 mg L^−1^ of organic acids, 0.91 mg L^−1^ of fatty acids and 314 mg L^−1^ of mineral nutrients [27]. Before harvesting, the total water potential was measured according to [27].

At the harvest, five replicate plants from each treatment were divided into leaves, stems and fruits and immediately weighed to give the fresh weight. The shoot weight was calculated as sum of leaves and stems. They were then oven-dried at 80 °C to constant weight (72 h) and the dry weight was measured. The other five replicate plant leaf samples were frozen in liquid nitrogen and stored at −80 °C. The frozen plant material was homogenized by a swing mill (MM 200, Retch, Haan, Germany) at 30 Hz for at least 1 min in order to obtain a fine powder. The metal balls (1 cm) and containers (stainless steel of 20 mL volume) were precooled in liquid nitrogen before homogenization. Then, the fine powder was transferred to several precooled 1.5 mL tubes (eppis) and stored at −80 °C. The measures were repeated on five or at least three different samples (biological replicates).

### 4.2. Ions, Metabolites and Antioxidants Analysis

Primary amino acids and proline extraction was performed by mixing 50 mg of powdered pooled leaves with 1 mL of ethanol:water in the ratio 2:3 (*v*/*v*), kept overnight at 4 °C and centrifuged at 14,000× *g* at 4 °C for 10 min. Amino acids were estimated by HPLC after pre-column derivatization by *o*-phthaldialdehyde (OPA) according to [79] using a 5 µm ZORBAX Eclipse Plus C18, 250 × 4.6 mm inte70% BNS al diameter; (Agilent Technologies Italia S.p.A, Cernusco sul Naviglio (MI), Italy) protected by a C18 Security Guard pre-column (4 × 3 mm ID; Phenomenex Inc., Torrance, CA, USA). Proline was determined according to [67]. Ions were extracted from 50 mg of powdered dried tissue suspended in 5 mL of MilliQ grade water (Milli-Q PLUS, Millipore, Burlington, MA, USA), and then subjected to three freeze–thaw cycles. After centrifugation, clear supernatants were assayed by ion-exchange chromatography using a DX500 apparatus and columns (Dionex, Sunnyvale, CA, USA). In particular, an IONPAC-ATC1 anion trap column, an IONPAC-AG11 guard column and an analytical IONPAC-AS11 4-mm column, fitted with an ASRSII 4-mm suppressor for anions, and an IONPAC-CTC cation trap column, an IONPAC-CG12A guard column (Dionex) and an analytical IONPAC-CS12A 4-mm column, fitted with a CSRS 4-mm suppressor for cations, coupled to a CD20 conductivity detector were used according to [80]. Soluble proteins, starch, and sugars were evaluated according to [68]; starch was expressed as glucose equivalents. Ascorbic acid (ASCAc), dehydroascorbic acid (DHA), and reduced and oxidized glutathione (GSH and GSSG) were extracted as described by [81] and [82], respectively, and determined according to [83]. The sap osmolality of leaves was obtained according to [84]. The contribution of ions and metabolites to osmolality was calculated according to [50,85].

### 4.3. Statistics, Heat Map and Principal Component Analysis

Analysis of variance (ANOVA) was conducted for all traits. Principal component analysis (PCA) was conducted using Minitab 18 statistical software (Minitab Inc., State College, PA, USA), according to [86]. The PCA outputs included variable loading to each selected component and treatment component scores [37].

## 5. Conclusions

The use of biostimulants may play an important role for improving nutrients and water use efficiency in sustainable agricultural systems. However, due to their complex nature, a deeper understanding of their mechanism of action is necessary in order to design biostimulants formulations that maximize their effects. Modulation of amino acids and potassium levels in MicroTom plants treated with *Ascophyllum nodosum* based biostimulants improved osmotic imbalance, control cell growth and expansion, improved nitrate uptake and limited cellular damages caused by ROS accumulation, all of which are critical under saline stress. The coordination of these mechanisms under stress adaptation can be mediated by a total reorganization of cellular and tissue levels of carbon and nitrogen metabolites that could arrest further growth and differentiation in young tissues while ensuring the achievement of adequate water and ion homeostasis. A plant’s ability to efficiently use available ions (sodium and potassium) and compatible solutes has to be orchestrated with energy saving metabolic adaptation. This can be achieved by using proper N storage forms (alanine instead of amides) associated with less expensive mechanism for the production of ATP and ROS scavenging. The use of these two biostimulants has also been instrumental to highlight the enormous versatility of the nitrogen metabolism that with relatively few specific changes can allow plant viability also in conditions of salinity/osmotic stress and nutritional restriction. While our results show promise in understanding how these biostimulants affect nitrogen metabolism and salt stress tolerance, it remains unclear if these findings will easily translate into widely grown commercial varieties. If biostimulant formulations can be tailored to specific phenotypes and outcomes, their utility will greatly increase. However, in order to achieve tunable and customized formulations, it is essential to understand the underlying interactions and mechanisms. Future research must seek to interpret these results in commercial field crops in order to confirm the mechanism of action of biostimulants at large and exploit/potentiate them to improve their function in diverse agricultural contexts

## Figures and Tables

**Table 1:** Significance of the main factors (nutrient solution, biostimulant and salinity) and their interactions on leaves fresh and dry weight, shoot fresh weight, plant height, fruit fresh weight and dry weight and number of fruits. MicroTom plants were harvested at 90 days after sowing (DAS) after being fertigated since 35 DAS with a 100% or 70% basic nutrient solution (BNS), in combination with two different commercial *Ascophyllum nodosum* extracts, Rygex at 2.5 mL L^−1^ and Superfifty (SF) at 2.0 mL L^−1^ and three salinity levels of 0 (S0), 42.5 (S1), and 85 (S2) mM NaCl. Control was without biostimulant.

	Leaves FW	Leaves DW	Fruit FW	Fruit DW	Number of Fruits
	g	g	g	g	
**Nutrient solution (N)**	ns	ns	ns	ns	ns
100% BNS	13.96	1.94	31.19	2.66	15.64
70% BNS	13.98	1.80	33.63	2.81	15.80
**Biostimulant (B)**	ns	ns	**	ns	ns
Control	14.75	2.00	34.69 a	2.90	16.30
Rygex	13.43	1.73	28.78 b	2.48	14.60
Superfifty	13.74	1.89	33.76 a	2.82	16.27
**Salinity (S)**	*	ns	***	***	***
S0	15.23 a	1.95	43.57 a	3.22 a	17.73 a
S1	14.62 ab	1.93	29.57 b	2.63 b	16.23 a
S2	12.08 b	1.74	24.09 c	2.35 b	13.20 b
**NxB**	ns	ns	ns	ns	ns
**NxS**	ns	ns	ns	ns	ns
**BxS**	ns	ns	ns	ns	ns
**NxBxS**	ns	ns	ns	ns	ns
100% Control S0	14.10	1.86	46.72	3.35	18.60
100% Control S1	17.72	2.32	27.07	2.15	16.00
100% Control S2	11.47	2.03	25.52	2.69	15.20
100% Rygex S0	14.28	1.81	34.14	2.68	17.00
100% Rygex S1	12.26	1.57	27.51	2.58	14.80
100% Rygex S2	10.84	1.52	21.54	2.17	11.60
100% Superfifty S0	15.39	2.10	39.25	3.03	16.40
100% Superfifty S1	15.43	2.33	32.47	2.56	17.00
100% Superfifty S2	14.19	1.96	26.45	2.72	14.20
70% Control S0	15.27	1.95	51.19	3.58	16.60
70% Control S1	14.21	1.67	29.92	2.99	16.40
70% Control S2	15.74	2.18	27.73	2.65	15.00
70% Rygex S0	15.14	1.83	42.99	3.27	19.80
70% Rygex S1	16.90	2.15	26.95	2.58	15.40
70% Rygex S2	11.19	1.51	19.53	1.62	9.00
70% Superfifty S0	17.17	2.14	47.12	3.42	18.00
70% Superfifty S1	11.20	1.53	33.49	2.94	17.80
70% Superfifty S2	9.06	1.25	23.75	2.23	14.20

ns, *, **, *** Nonsignificant or significant at *p* ≤ 0.05, 0.01, and 0.001, respectively. Different letters indicate statistically different groups (*p* < 0.05, Tukey’s post hoc test following ANOVA; *n* = 5 for morphological traits and *n* = 3 for ions measurements).

**Table 2:** Significance of the main factors (nutrient solution, biostimulant and salinity) and their interactions on ions content in leaves of MicroTom plants. Experimental design is as in Table 1.

	Calcium	Chloride	Potassium	Magnesium	Nitrate	Sodium	K^+^:Na^+^
	mg g^−1^ FW	mg g^−1^ FW	mg g^−1^ FW	mg g^−1^ FW	mg g^−1^ FW	mg g^−1^ FW	
**Nutrient solution (N)**	ns	ns	ns	***	*	***	ns
100% BNS	1.64	1.95	5.37	0.39 b	0.15 a	1.03 a	16.92
70% BNS	1.68	1.68	5.43	0.47 a	0.13 b	0.80 b	16.04
**Biostimulant (B)**	ns	***	ns	**	***	*	**
Control	1.57	2.11 a	5.24	0.39 b	0.12 b	0.85 b	17.48 a
Rygex	1.65	1.02 b	5.29	0.42 ab	0.14 b	1.00 a	12.79 b
Superfifty	1.76	2.33 a	5.66	0.47 a	0.17 a	0.92 ab	19.16 a
**Salinity (S)**	ns	***	***	ns	***	***	***
S0	1.63	0.78 c	6.29 a	0.45	0.17 a	0.17 c	39.47 a
S1	1.68	1.91 b	5.66 a	0.43	0.15 b	0.85 b	7.41 b
S2	1.67	2.76 a	4.25 b	0.41	0.10 c	1.74 a	2.54 c
**NxB**	**	ns	ns	ns	ns	ns	ns
**NxS**	ns	*	ns	ns	*	**	*
**BxS**	ns	**	ns	ns	**	*	**
**NxBxS**	ns	ns	ns	ns	ns	*	*
100% Control S0	1.93	1.17	6.18	0.46	0.15	0.15 g	42.26 ab
100% Control S1	1.88	3.24	6.07	0.34	0.13	1.13 cde	5.40 d
100% Control S2	1.77	3.11	3.84	0.30	0.11	1.82 ab	2.13 d
100% Rygex S0	0.96	0.56	5.91	0.33	0.21	0.19 g	32.22 b
100% Rygex S1	1.65	1.30	5.60	0.43	0.15	1.05 cdef	5.39 d
100% Rygex S2	1.72	1.07	4.17	0.38	0.06	2.13 ab	2.00 d
100% Superfifty S0	1.43	0.93	6.80	0.42	0.22	0.13 g	54.47 ab
100% Superfifty S1	1.70	2.71	5.82	0.43	0.16	0.92 def	6.24 d
100% Superfifty S2	1.74	3.51	3.91	0.40	0.14	1.82 ab	2.15 d
70% Control S0	1.26	0.83	6.06	0.44	0.13	0.14 g	43.60 ab
70% Control S1	1.38	1.64	4.97	0.42	0.11	0.63 efg	7.87 d
70% Control S2	1.19	2.68	4.34	0.40	0.08	1.20 cd	3.62 d
70% Rygex S0	1.81	0.51	6.32	0.47	0.17	0.23 g	28.30 bc
70% Rygex S1	1.76	1.28	4.89	0.46	0.14	0.87 def	5.68 d
70% Rygex S2	2.01	1.39	4.84	0.48	0.08	1.53 bc	3.15 d
70% Superfifty S0	2.39	0.71	6.45	0.61	0.14	0.18 g	36.00 b
70% Superfifty S1	1.71	1.31	6.59	0.48	0.20	0.50 fg	13.88 cd
70% Superfifty S2	1.58	4.79	4.42	0.50	0.14	1.95 ab	2.21 d

ns, *, **, *** Nonsignificant or significant at *p* ≤ 0.05, 0.01, and 0.001, respectively. Different letters indicate statistically different groups (*p* < 0.05, Tukey’s post hoc test following ANOVA; *n* = 3).

**Table 3:** Significance of the main factors (nutrient solution, biostimulant and salinity) and their interactions on content of ascorbate (Asc), Dehydroascorbate (DHA), DHA:Asc, reduced glutathione (GSH), oxidized glutathione (GSSG) and GSSG:GSH in leaves of MicroTom plants. Experimental design is as in Table 1.

	Asc	DHA	DHA/Asc	Glut Rid	GSSG	GSSG:GSH
	nmol g^−1^ FW	nmol g^−1^ FW		nmol g^−1^ FW	nmol g^−1^ FW	
**Nutrient solution (N)**	ns	**	***	*	ns	ns
100% BNS	9.30	2.68 a	0.31 a	242.91 a	148.01	0.66
70% BNS	10.10	2.13 b	0.22 b	223.87 b	140.41	0.67
**Biostimulant (B)**	*	***	***	***	ns	***
Control	9.95 ab	1.60 b	0.17 b	204.19 b	152.78	0.75 a
Rygex	10.53 a	1.72 b	0.17 b	175.87 c	138.71	0.79 a
Superfifty	8.62 b	3.90 a	0.45 a	320.11 a	141.14	0.44 b
**Salinity (S)**	ns	***	***	**	ns	*
S0	9.04	3.02 a	0.35 a	257.69 a	147.71	0.62 b
S1	10.54	2.06 b	0.21 b	225.02 b	141.15	0.67 ab
S2	9.52	2.12 b	0.23 b	217.45 b	143.78	0.70 a
**NxB**	ns	***	***	*	ns	ns
**NxS**	ns	ns	ns	ns	ns	ns
**BxS**	ns	***	***	ns	*	ns
**NxBxS**	ns	ns	ns	ns	ns	ns
100% Control S0	8.08	1.62	0.20	207.9	146.4	0.70
100% Control S1	11.05	1.55	0.15	211.2	158.6	0.75
100% Control S2	9.71	1.56	0.17	178.7	131.9	0.74
100% Rygex S0	8.91	1.59	0.18	180.6	133.7	0.74
100% Rygex S1	11.57	1.63	0.15	193.6	154.7	0.81
100% Rygex S2	8.93	1.55	0.18	172.0	148.7	0.84
100% Superfifty S0	8.47	6.27	0.74	386.1	136.5	0.35
100% Superfifty S1	8.05	4.27	0.52	295.1	131.3	0.46
100% Superfifty S2	8.91	4.04	0.45	361.0	190.3	0.53
70% Control S0	9.12	1.61	0.18	227.2	161.9	0.71
70% Control S1	12.42	1.64	0.13	230.7	182.2	0.79
70% Control S2	9.32	1.58	0.17	169.4	135.7	0.80
70% Rygex S0	10.78	2.18	0.22	216.6	157.4	0.74
70% Rygex S1	11.32	1.71	0.16	137.2	117.9	0.86
70% Rygex S2	11.65	1.64	0.14	155.1	119.9	0.77
70% Superfifty S0	8.90	4.88	0.55	327.7	150.4	0.46
70% Superfifty S1	8.86	1.57	0.18	282.3	102.2	0.35
70% Superfifty S2	8.57	2.36	0.27	268.5	136.2	0.51

ns, *, **, *** Nonsignificant or significant at *p* ≤ 0.05, 0.01, and 0.001, respectively. Different letters indicate statistically different groups (*p* < 0.05, Tukey’s post hoc test following ANOVA; *n* = 3).

**Table 4:** Significance of the main factors (nutrient solution, biostimulant and salinity) and their interactions on content of glucose, fructose, sucrose, starch, hexoses and soluble proteins in leaves of MicroTom plants. Experimental design is as in Table 1.

	Glucose	Fructose	Hexoses	Sucrose	Starch	Soluble Proteins
	µmol g^−1^ FW	µmol g^−1^ FW	µmol g^−1^ FW	µmol g^−1^ FW	µmol g^−1^ Glu_eq_ FW	mg g^−1^ FW
**Nutrient solution (N)**	ns	*	ns	ns	ns	ns
100% BNS	10.17	2.56 a	12.73	2.05	2.76	12.24
70% BNS	10.70	2.25 b	12.47	2.26	2.76	12.21
**Biostimulant (B)**	***	ns	***	***	***	**
Control	13.73 a	2.38	16.11 a	3.04 a	3.22 a	13.42 a
Rygex	9.46 b	2.38	11.85 b	1.82 b	2.26 c	12.32 a
Superfifty	7.95 c	2.47	9.84 c	1.57 b	2.80 b	10.89 b
**Salinity (S)**	**	**	**	*	**	ns
S0	10.71 a	2.05 b	12.75 b	1.92 b	2.53 b	12.22
S1	11.60 a	2.68 a	13.49 a	2.22 ab	3.11 a	12.31
S2	9.05 b	2.51 a	11.55 b	2.33 a	2.65 b	12.14
**NxB**	***	ns	***	*	ns	ns
**NxS**	ns	ns	ns	*	*	ns
**BxS**	*	ns	ns	ns	***	ns
**NxBxS**	***	ns	***	***	***	**
100% Control S0	17.68 a	2.19	19.87 a	3.54 ab	3.61 ab	14.40 ab
100% Control S1	16.87 ab	2.43	19.30 a	2.66 bcd	3.42 b	13.46 ab
100% Control S2	9.60 cde	2.76	12.35 bcd	2.44 bcde	3.07 b	12.23 ab
100% Rygex S0	6.46 e	1.85	8.31 d	1.15 e	2.47 bc	11.19 ab
100% Rygex S1	7.17 e	2.66	9.83 cd	1.58 de	2.22 bc	12.39 ab
100% Rygex S2	8.15 de	2.73	10.88 bcd	1.93 de	2.03 bc	12.71 ab
100% Superfifty S0	8.20 de	2.60	10.80 bcd	1.45 de	2.46 bc	10.87 ab
100% Superfifty S1	8.54 de	2.93	11.48 bcd	1.86 de	2.91 b	11.27 ab
100% Superfifty S2	8.86 de	2.87	11.73 bcd	1.84 de	2.67 bc	11.64 ab
70% Control S0	9.45 cde	2.06	11.51 bcd	1.96 cde	1.16 c	10.61 ab
70% Control S1	15.72 ab	2.69	18.41 a	3.33 abc	5.18 a	13.92 ab
70% Control S2	13.08 abcd	2.12	15.20 abc	4.29 a	2.87 b	15.90 a
70% Rygex S0	14.98 abc	1.71	16.69 ab	2.21 bcde	2.33 bc	14.52 ab
70% Rygex S1	11.93 bcde	2.59	14.51 abc	2.16 bcde	2.05 bc	12.05 ab
70% Rygex S2	8.10 de	2.75	10.85 bcd	1.90 de	2.49 bc	11.08 ab
70% Superfifty S0	7.46 de	1.89	9.35 cd	1.20 e	3.15 b	11.74 ab
70% Superfifty S1	8.26 de	2.81	11.07 bcd	1.50 de	2.79 bc	10.41 ab
70% Superfifty S2	6.49 e	1.81	8.30 d	1.57 de	2.80 bc	9.29 b

ns, *, **, *** Nonsignificant or significant at *p* ≤ 0.05, 0.01, and 0.001, respectively. Different letters indicate statistically different groups (*p* < 0.05, Tukey’s post hoc test following ANOVA; *n* = 3).

**Table 5:** Significance of the main factors and their interactions on content of leaf free amino acids of MicroTom plants. Experimental design is as in Table 1.

	Alanine	Asparagine	GABA	Glutamine	Glutamate	Glycine	Proline	Serine	Threonine	Minor AA	Total AA	Gln:Glu
	µmol g^−1^ FW	µmol g^−1^ FW	µmol g^−1^ FW	µmol g^−1^ FW	µmol g^−1^ FW	µmol g^−1^ FW	µmol g^−1^ FW	µmol g^−1^ FW	µmol g^−1^ FW	µmol g^−1^ FW	µmol g^−1^ FW	
**Nutrient solution (N)**	*	***	***	***	*	***	***	***	***	***	***	***
100% BNS	1.01 a	0.88 a	2.39 a	24.64 a	1.94 a	0.39 a	0.58 a	0.71 a	0.34 a	4.82 a	37.47 a	13.38 a
70% BNS	0.91 b	0.76 b	1.88 b	11.74 b	1.76 b	0.30 b	0.43 b	0.57 b	0.27 b	3.90 b	21.99 b	6.93 b
**Biostimulant (B)**	***	ns	***	ns	**	***	***	***	***	***	***	ns
Control	0.64 b	0.85	2.30 b	17.57	1.76 b	0.17 b	0.39 b	0.43 b	0.19 b	3.20 b	26.82 b	9.97
Rygex	0.61 b	0.78	1.42 c	17.90	1.76 b	0.20 a	0.57 a	0.46 b	0.19 b	2.52 c	26.67 b	11.24
Superfifty	1.65 a	0.83	2.70 a	19.35	2.06 a	0.68 a	0.55 a	1.04 a	0.54 a	7.37 a	36.16 a	9.33
**Salinity (S)**	*	ns	ns	***	*	ns	***	ns	*	ns	***	***
S0	1.02 a	0.86	2.14	15.11 b	1.99 a	0.36	0.32 a	0.64	0.33 a	4.46	26.76 b	7.44 c
S1	0.89 b	0.79	1.96	16.39 b	1.79 b	0.32	0.50 b	0.61	0.28 b	4.08	27.38 b	9.43 b
S2	0.97 a	0.81	2.30	23.22 a	1.78 b	0.35	0.69 c	0.67	0.30 b	4.54	35.22 a	13.67 a
**NxB**	***	***	*	**	***	***	ns	*	ns	***	**	***
**NxS**	ns	ns	ns	ns	ns	*	***	ns	ns	ns	ns	ns
**BxS**	*	***	**	***	***	**	ns	*	*	***	***	**
**NxBxS**	*	***	*	**	*	ns	***	ns	ns	*	*	*
100% Control S0	0.94 b	1.04 abc	2.67 abcde	20.03 cdef	2.22 ab	0.32	0.28 fg	0.65	0.31	4.12 d	32.05 cde	9.21 bcd
100% Control S1	0.63 bc	1.02 abcd	2.70 abcd	12.91 efg	1.87 bc	0.19	0.57 cd	0.47	0.20	3.36 def	22.77 efgh	7.01 cd
100% Control S2	0.79 bc	1.10 a	2.84 abc	32.16 a	2.02 abc	0.15	0.57 cd	0.50	0.17	3.95 de	43.93 ab	16.18 b
100% Rygex S0	0.54 c	0.69 efg	1.26 f	13.59 efg	1.56 bcd	0.16	0.30 efg	0.43	0.20	2.30 fg	21.67 efgh	8.93 bcd
100% Rygex S1	0.59 c	0.72 defg	1.47 cdef	26.99 abc	1.67 bcd	0.20	0.69 bc	0.49	0.19	2.50 fg	36.22 bcd	16.07 b
100% Rygex S2	0.61 c	0.74 cdef	1.65 bcdef	33.13 a	1.44 cd	0.23	0.91 ab	0.51	0.20	2.84 efg	42.99 ab	24.95 a
100% Superfifty S0	1.71 a	1.08 ab	3.40 a	31.29 ab	2.67 a	0.79	0.29 gf	1.12	0.64	8.09 ab	50.23 a	11.95 bcd
100% Superfifty S1	1.51 a	0.75 cdef	2.73 abcd	24.13 abcd	1.92 abc	0.74	0.57 cd	1.06	0.57	8.20 a	41.74 abc	13.09 bc
100% Superfifty S2	1.74 a	0.75 cdef	2.82 abc	27.49 abc	2.11 abc	0.71	1.03 a	1.13	0.55	8.02 ab	45.59 ab	13.06 bc
70% Control S0	0.52 c	0.94 abcde	1.50 cdef	11.61 fg	1.81 bc	0.11	0.18 g	0.35	0.18	2.50 fg	19.18 fgh	6.56 cd
70% Control S1	0.48 c	0.42 g	1.13 f	6.81 g	1.02 d	0.08	0.29 efg	0.20	0.08	2.02 g	12.10 h	6.99 cd
70% Control S2	0.51 c	0.60 fg	2.98 ab	21.91 bcde	1.60 bcd	0.15	0.48 cdef	0.43	0.19	3.25 def	30.89 cde	13.88 bc
70% Rygex S0	0.59 c	0.61 fg	1.37 def	6.14 g	1.70 bcd	0.16	0.51 cdef	0.36	0.16	1.98 g	13.35 gh	3.90 d
70% Rygex S1	0.64 bc	1.01 abcd	1.50 cdef	12.95 efg	2.28 ab	0.21	0.39 defg	0.51	0.21	2.84 efg	22.33 efgh	5.93 cd
70% Rygex S2	0.67 bc	0.90 abcdef	1.31 ef	14.61 defg	1.90 bc	0.22	0.63 cd	0.46	0.16	2.64 fg	23.46 efg	7.69 cd
70% Superfifty S0	1.80 a	0.78 bcdef	2.65 abcde	8.02 g	1.99 abc	0.62	0.38 defg	0.93	0.50	7.80 ab	24.05 efg	4.09 d
70% Superfifty S1	1.66 a	0.84 abcdef	2.31 abcdef	14.11 efg	2.03 abc	0.58	0.45 cdef	1.00	0.50	5.55 bc	29.55 def	7.05 cd
70% Superfifty S2	1.48 a	0.75 cdef	2.22 abcdef	10.03 g	1.61 bcd	0.62	0.55 cde	0.99	0.49	6.56 c	24.45 ef	6.29 cd

ns, *, **, *** Nonsignificant or significant at *p* ≤ 0.05, 0.01, and 0.001, respectively. Different letters indicate statistically different groups (*p* < 0.05, Tukey’s post hoc test following ANOVA; *n* = 3).

**Table 6:** Leaf osmolality and relative contribution (%) of inorganic ions, soluble sugars, amino acids and other metabolites towards the total osmolality of MicroTom plants. Experimental design is as in Table 1. The S.D. was lower than 15.8% of the average value.

A. 100% BNS	Control			Rygex			Superfifty		
	S0	S1	S2	S0	S1	S2	S0	S1	S2
**Osmolality (mosmol/kg)**	**349**	**495**	**497**	**300**	**361**	**504**	**323**	**448**	**576**
Chloride	9.5	18.5	17.6	5.3	10.1	6	8.1	17.1	17.2
Potassium	45.3	31.4	19.7	50.5	39.7	21.1	53.8	33.2	17.4
Sodium	1.9	10	15.9	2.7	12.6	18.4	1.7	8.9	13.7
**Measured ions**	**56.7**	**59.8**	**53.2**	**58.4**	**62.4**	**45.5**	**63.6**	**59.2**	**48.2**
Glucose	17.7	16.9	9.6	6.5	7.2	8.1	8.2	8.5	8.9
Fructose	2.2	2.4	2.8	1.8	2.7	2.7	2.6	2.9	2.9
Sucrose	3.5	2.7	2.4	1.1	1.6	1.9	1.5	1.9	1.8
***Sum of soluble sugars***	***23.4***	***22***	***14.8***	***9.5***	***11.4***	***12.8***	***12.2***	***13.3***	***13.6***
Ala	0.3	0.1	0.2	0.2	0.2	0.1	0.5	0.3	0.3
Asn	0.6	0.3	0.5	0.6	0.6	0.5	0.8	0.5	0.4
Asp	0.3	0.2	0.2	0.2	0.2	0.1	0.3	0.2	0.1
GABA	0.4	0.3	0.3	1.1	0.8	0.6	0.8	0.6	0.5
Gln	5.7	2.6	6.5	4.5	7.5	6.6	9.7	5.4	4.8
Glu	0.6	0.4	0.4	0.5	0.5	0.3	0.8	0.4	0.4
Pro	0.1	0.1	0.1	0.1	0.2	0.2	0.1	0.1	0.2
Minor AA	1.2	0.7	0.8	0.8	0.7	0.6	2.5	1.8	1.4
***Total amino acids***	***9.2***	***4.6***	***8.8***	***7.2***	***10***	***8.5***	***15.6***	***9.3***	***7.9***
**Measured organic osmolytes**	**32.6**	**26.6**	**23.6**	**16.7**	**21.4**	**21.3**	**27.8**	**22.7**	**21.5**
**Other metabolites**	**10.7**	**13.6**	**23.2**	**24.9**	**16.1**	**33.2**	**8.6**	**18.1**	**30.3**
**B. 70% BNS**	**Control**			**Rygex**			**Superfifty**		
	**S0**	**S1**	**S2**	**S0**	**S1**	**S2**	**S0**	**S1**	**S2**
**Osmolality (mosmol/kg)**	**418**	**551**	**655**	**289**	**373**	**439**	**360**	**435**	**572**
Chloride	5.6	8.4	11.5	5	9.7	8.9	5.6	8.5	23.6
Potassium	37	23.1	16.9	55.9	33.5	28.2	45.8	38.7	19.8
Sodium	1.5	5	8	3.4	10.1	15.2	2.2	5	14.8
**Measured ions**	**44.1**	**36.5**	**36.5**	**64.4**	**53.3**	**52.3**	**53.6**	**52.3**	**58.2**
Glucose	9.5	15.7	13.1	15	11.9	8.1	7.5	8.3	6.5
Fructose	2.1	2.7	2.1	1.7	2.6	2.8	1.9	2.8	1.8
Sucrose	2	3.3	4.3	2.2	2.2	1.9	1.2	1.5	1.6
***Sum of soluble sugars***	***13.5***	***21.7***	***19.5***	***18.9***	***16.7***	***12.8***	***10.6***	***12.6***	***9.9***
Ala	0.1	0.1	0.1	0.2	0.2	0.2	0.5	0.4	0.3
Asn	0.2	0.1	0.3	0.4	0.3	0.3	0.3	0.3	0.2
Asp	0.2	0.1	0.1	0.2	0.3	0.2	0.2	0.2	0.1
GABA	0.4	0.2	0.5	0.5	0.4	0.3	0.7	0.5	0.4
Gln	2.8	1.2	3.3	2.1	3.5	3.3	2.2	3.2	1.8
Glu	0.4	0.2	0.2	0.6	0.6	0.4	0.6	0.5	0.3
Pro	0	0.1	0.1	0.2	0.1	0.1	0.1	0.1	0.1
Minor AA	0.6	0.4	0.5	0.7	0.8	0.6	2.2	1.3	1.1
***Total amino acids***	***4.6***	***2.2***	***4.7***	***4.6***	***6***	***5.3***	***6.7***	***6.8***	***4.3***
**Measured organic osmolytes**	**18.1**	**23.9**	**24.2**	**23.5**	**22.7**	**18.1**	**17.2**	**19.4**	**14.1**
**Other metabolites**	**37.8**	**39.6**	**39.3**	**12.1**	**24**	**29.6**	**29.2**	**28.4**	**27.6**

## Data Availability

Not applicable.

## Supplementary Materials

The following are available online at https://www.mdpi.com/article/10.3390/plants10061044/s1, Figure S1: Leaf content of starch, soluble proteins, hexoses, sucrose, dehydroascorbate to ascorbate ratio (DHA:Asc) and oxidized to reduced glutathione ratio (GSSG:GSH). Bar colors and experimental design are as in Figure 1. The values are mean ± S.D. (*n* = 3). Significance of the main factors (nutrient solution, biostimulant and salinity) and their interactions is shown in Table 3 and Table 4.
