# Supplementary material for: Ascophyllum nodosum Based Extracts Counteract Salinity Stress in Tomato by Remodeling Leaf Nitrogen Metabolism
Source: Plants (Basel). 2021 May 21;10(6):1044. doi: 10.3390/plants10061044 (PMC8224312; doi:10.3390/plants10061044)
Supplement: Supplementary file 1 [file plants-10-01044-s001.zip › plants-1226829-supplementary.pdf]

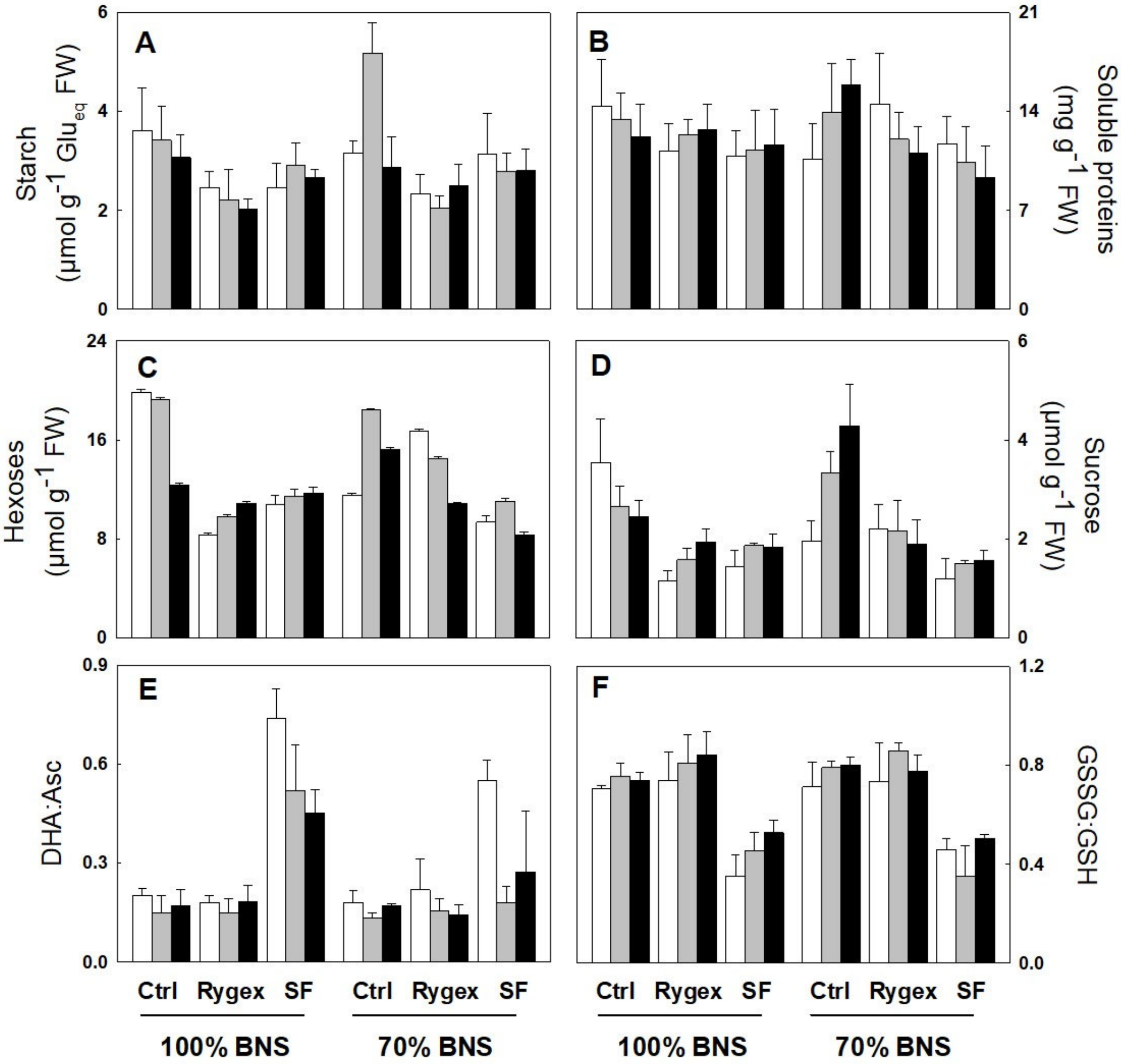

Supplemental figure 1. Leaf content of starch (A), soluble proteins (B), hexoses (C), sucrose (D) dehydroascorbate to ascorbate ratio (DHA:Asc) (E) and oxidized to reduced glutathione ratio (GSSG:GSH) (F). Bar colors and experimental design are as in Figure 1. The values are mean ± S.D. (n = 3). Significance of the main factors (nutrient solution, biostimulant and salinity) and their interactions is shown in Tables 3 and 4.
